# Improvements in anthropometric measures and gastrointestinal tolerance in patients with cystic fibrosis by using a digestive enzyme cartridge with overnight enteral nutrition

**DOI:** 10.1002/ncp.10831

**Published:** 2022-02-24

**Authors:** Sara J. Hendrix, Patrick A. Flume, Eric R. First, Albert Archie Stone, Mark Van Buskirk

**Affiliations:** ^1^ Medical University of South Carolina Charleston South Carolina USA; ^2^ Alcresta Therapeutics, Inc Newton MA USA; ^3^ Data Reduction LLC Chester New Jersey USA

**Keywords:** cystic fibrosis, enteral nutrition, exocrine pancreatic insufficiency, gastrointestinal symptoms, lipase, pancreatic enzymes

## Abstract

**Background:**

Patients with cystic fibrosis (CF) and pancreatic insufficiency are at risk for suboptimal fat absorption, inability to maintain weight, poor growth, and increased gastrointestinal (GI) symptoms due to malabsorption. Enteral nutrition (EN) is used to supplement caloric intake and requires pancreatic enzyme replacement for effective digestion. We evaluated the relationship between long‐term use of an in‐line digestive enzyme cartridge with EN and changes in anthropometric measures and GI symptoms in patients with CF.

**Methods:**

This is a single‐center, retrospective case review of patients with CF using a digestive enzyme cartridge with EN. Data were collected from the patient medical records and included weight, height, body mass index, EN regimen, and reported GI symptoms.

**Results:**

Thirteen pediatric and five adult patients with a mean age of 12.6 years used a digestive enzyme cartridge with EN for a period of 3–27 months. Most patients (*n* = 14) had been using oral digestive enzymes with EN before using the digestive enzyme cartridge, whereas four started from the onset of EN. The indications to convert from oral enzymes to the digestive enzyme cartridge included poor growth (72.2%) and poor tolerance of EN (69.2%). There was a reduction in reported GI symptoms after initiating use of the digestive enzyme cartridge. After 12 months of digestive cartridge use, there were improvements in anthropometrics.

**Conclusions:**

This real‐world experience with prolonged use of a digestive enzyme cartridge with EN demonstrated improved clinical outcomes and a reduction in GI symptoms in patients with CF.

## INTRODUCTION

Patients with cystic fibrosis (CF) and exocrine pancreatic insufficiency (EPI) frequently experience growth failure and malnutrition due to the inadequate digestion of nutrients and, consequently, malabsorption, increased energy needs, and reduced appetite. Addressing nutrient deficit and ameliorating maldigestion and malabsorption are essential to achieve normal growth to support optimal pulmonary function and to prolong life.^1^, ^2^ The Cystic Fibrosis Foundation (CFF) Guidelines recommend that children from birth to 2 years of age maintain a weight‐for‐length (WFL) at or above the 50th percentile and children and adolescents aged 2–20 years maintain a body mass index (BMI; weight in kilograms divided by height in meters squared) at or above the 50th percentile. The weight target for adults is a BMI at or above 22 for women and 23 for men, as studies demonstrate a clear connection with improved pulmonary function when adequate weight is achieved.^3^ EPI is characterized by a deficiency of exocrine pancreatic enzymes, resulting in the inability to properly digest fat, carbohydrate, and protein.^4^ Gastrointestinal (GI) symptoms of fat malabsorption can result in steatorrhea, diarrhea, abdominal pain, nausea, bloating, and decreased appetite.^5^ Patients with EPI use oral pancreatic enzyme replacement therapy (PERT) in conjunction with meals to increase the absorption of fat and other nutrients.^6^ Despite the use of PERT, there are patients who fail to meet their nutrition goals and require supplemental nutrition, and for patients with moderate to severe malnutrition, the recommendation is to consider enteral nutrition (EN) support to supplement oral intake.^7^ Over 10% of people with CF use supplemental EN.^8^

There is a lack of clinical trial data in patients with EPI who require EN. Oral PERT is designed to be taken before a meal or bolus feed, and activity is estimated to last ∼45–60 min after administration.^9^ On the basis of best‐practice experience, it has been commonplace for people with CF who are receiving EN to take enzymes orally at the start and end of nocturnal feeds; however, this leaves several hours without enzymatic coverage. Mixing PERT into EN formula is not supported by guidelines, and this route is associated with risks such as feeding tube obstruction and inconsistent enzyme dosing.^10^ Although several options exist to deliver protein in predigested forms, there are no formula options that contain predigested triglycerides because the fatty acid products of normal digestion are liable to oxidation and are not shelf stable.^11^ To address these challenges, a single‐use digestive enzyme cartridge that connects in‐line with the enteral feeding set was developed. The digestive cartridge has only lipase and achieves >90% hydrolysis of triglycerides in most formulas tested in vitro and has been approved for use in patients with fat malabsorption who are 5 years and older.^12^ A multicenter, randomized, double‐anonymized, crossover trial concluded that short‐term use of the digestive cartridge was safe and well tolerated in children and adults with CF and resulted in significantly increased levels of plasma ω‐3 fatty acids when used in conjunction with EN.^13^ A longer‐term, 90‐day, open‐label study further supported these findings.^14^ The cartridge contains lipase, which is covalently bound to small beads. As enteral formula flows through the cartridge, the lipase hydrolyzes fat to allow for the delivery of absorbable fatty acids and monoglycerides to the patient.^12^ Given the mechanism of action, we felt this should have been equally efficacious and safe in children younger than the label of 5 years. This study examines the use of a digestive enzyme cartridge for a period of 12 months in patients with CF and EPI who have been prescribed EN.

## METHODS

This is a single‐center, retrospective case review of patients with CF using a digestive enzyme cartridge with EN. Patients between the ages of 2 months and 35 years who have CF and EPI and have been prescribed EN with digestive enzyme cartridge were included in the analysis. The time frame of our analysis was between July 2016 and September 2018. The patients were seen at the accredited CF center by the treating physician and dietitian at ∼3‐month intervals. Baseline data, defined as patient status most prior to use of the digestive enzyme cartridge, were abstracted from the patient's medical records to include age, sex, anthropometrics, GI symptoms, indication for use of digestive enzyme cartridge, pancreatic enzyme regimen prior to starting the digestive enzyme cartridge, EN formula brand and daily volume, and serum vitamin D levels. Anthropometrics, general compliance with digestive enzyme cartridge use, and GI complaints were abstracted from the medical record for each clinic visit for up to 12 months after initiating the use of the digestive enzyme cartridge. Throughout the period of digestive enzyme cartridge use with EN, patients continued established medication regimens and received their usual oral diet. This project was determined to be for quality improvement and is therefore not subject to review or approval by an institutional review board.

## STATISTICAL METHODS

WFL percentiles, BMI percentiles, length‐for‐age (LFA) percentiles, stature‐for‐age (SFA) percentiles, *z*‐scores, and BMI were used to interpret growth measurements. World Health Organization (WHO) Child Growth Standards^15^ were used for patients <2 years of age, and Centers for Disease Control and Prevention (CDC) growth charts^16^ were used for patients 2–17 years of age. We determined change in WFL, BMI, LFA, and SFA percentiles and *z*‐scores for pediatric patients and change in BMI for adult patients from baseline to 3, 6, 9, and 12 months after initiation of the digestive enzyme cartridge. Within‐patient changes in efficacy measures from baseline to these intervals used either a one‐sample *t*‐test or Wilcoxon signed rank test, depending on whether the data were normally distributed. The recorded weight measurement closest to the identified time point was used. A supportive analysis used all available patient data to compute the change in efficacy measure over time (slope), expressed as the change per month (SAS, version 9.4). Other extracted data were summarized descriptively.

## RESULTS

Eighteen patients were included in the medical record review, including pediatric (*n* = 13) and adult (*n* = 5) patients. The characteristics at baseline and after initiation of the digestive enzyme cartridge are shown in Table 1. The mean (SD) age at the time of initiation of digestive enzyme cartridge use was 12.6 (9.5) years. The mean (SD) age of known GI tube placement was 10.3 (9.4) years. Fourteen (77.8%) patients were receiving PERT with EN before switching to the digestive enzyme cartridge. The average duration of time these patients were receiving EN before use of the digestive enzyme cartridge was 28.1 (32.2) months and was 17.8 (9.9) months while using the digestive enzyme cartridge. The mean percentage of energy requirements provided by EN was 50%.

**Table 1:** Patient characteristics at baseline and after initiation of a digestive enzyme cartridge

Characteristics	Data
Sex, *n* (%)	
Male	12 (66.7)
Female	6 (33.3)
Age at start of digestive enzyme cartridge use, years	
Mean (SD)	12.6 (9.5)
Min, Max	0.2, 35.6
Age at time of G‐tube placement, years	
Mean (SD)	10.3 (9.4)
Min, Max	0.3, 35.6
Age at start of digestive enzyme cartridge use, *n* (%)	
Birth to 2 years	2 (11)
>2–5 years	2 (11)
>5–12 years	7 (40)
>12–18 years	2 (11)
>18 years	5 (28)
Duration of EN before initiation of digestive enzyme cartridge, months	
Mean (SD)	28 (32)
Min, Max	0.0, 90
Duration of digestive enzyme cartridge use, months	
Mean, (SD)	18 (10)
Min, Max	3.0, 27
Indication for digestive enzyme cartridge, *n* (%)	
Poor growth	13 (72.2)

Abbreviations: EN, enteral nutrition; G‐tube, gastrostomy tube; Max, maximum; Min, minimum.

Five patients discontinued digestive enzyme cartridge use before 12 months. One patient discontinued digestive enzyme cartridge use because of issues with the cartridge disconnecting during overnight feeds; two patients were able to maintain a healthy weight without EN and therefore stopped using EN and the digestive enzyme cartridge; one patient moved away and was lost to follow‐up; and there was one death in a patient who had end‐stage lung disease. Data for these patients are included in the medical record review up to the time of stopping use of digestive enzyme cartridge or were lost to follow‐up.

GI adverse events were collected from verbatim responses in the medical records. Fourteen (77.8%) patients had GI complaints before starting the digestive enzyme cartridge; five patients (27.8%) reported GI complaints after initiating use of the digestive enzyme cartridge (Table 2). The most common complaints identified by pediatric patients were nausea/vomiting and abdominal pain, which were reduced by 75% and 33%, respectively, with cartridge use compared with baseline. For adults, nausea/vomiting was the most common complaint at baseline, which was reduced by 50% after initiating use of the enzyme cartridge.

**Table 2:** GI complaints before and after initiation of a digestive enzyme cartridge

**All (*N* = 18)**	**Precartridge condition, %**	**Using cartridge, %**
GI complaints (any type)	77.8	27.8
Abdominal pain	16.7	11.1
Nausea/vomiting	33.3	11.1

Pediatric (*n* = 13)	Precartridge condition	Using cartridge
GI complaints (any type)	76.9	23.1
Abdominal pain	23.1	15.4
Nausea/vomiting	30.8	7.7

Adults (*n* = 5)	Precartridge condition	Using cartridge
GI complaints (any type)	80.0	40.0
Abdominal pain	0	0
Nausea/vomiting	40.0	20.0

Abbreviation: GI, gastrointestinal.

**Figure 1:**
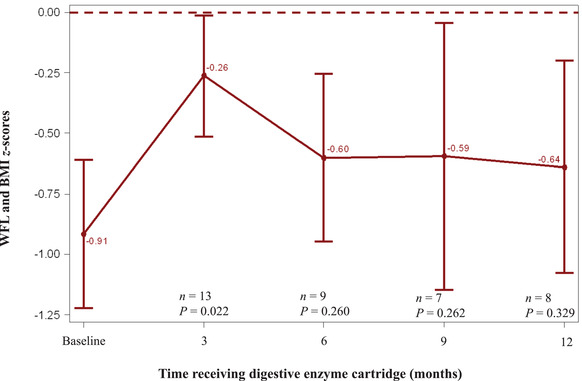
WFL and BMI *z*‐scores at baseline and 3‐month intervals after initiation of the digestive enzyme cartridge, mean (±SE). BMI, body mass index; WFL, weight‐for‐length

**Figure 2:**
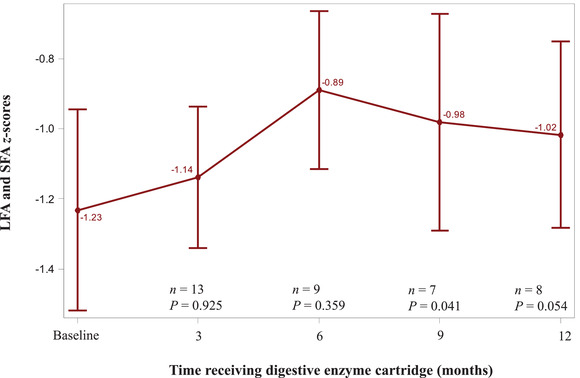
LFA and SFA *z*‐scores at baseline and 3‐month intervals after initiation of the digestive enzyme cartridge, mean (±SE). LFA, length‐for‐age; SFA, stature‐for‐age

**Figure 3:**
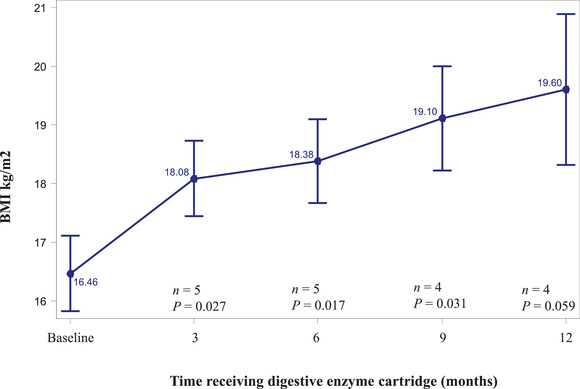
BMI at baseline and 3‐month intervals after initiation of the digestive enzyme cartridge, mean (±SE). BMI, body mass index

Among pediatric patients, mean WFL (for age of <24 months) and BMI (for age of 2–17 years) *z*‐scores improved from baseline at each subsequent follow‐up visit, although changes were only significant at the month 3 visit, with improvement from −0.91 to −0.26 (*P* = 0.022) as shown in Figure 1. Mean LFA (for age <24 months) and SFA (for age 2–17 years) *z*‐scores also improved from baseline to each follow‐up visit but were only significant at month 9, with improvement from −1.23 to −0.98 (*P* = 0.041) as shown in Figure 2. For adult patients, improvements in BMI from baseline (16.46; SD, 1.443) were seen at month 3 (18.08; SD, 1.436; *P* = 0.027), month 6 (18.38; SD, 1.602; *P* = 0.017), and month 9 (19.10; SD, 1.781; *P* = 0.031) as shown in Figure 3.

Vitamin D levels before and after initiation of the digestive enzyme cartridge were evaluated, comparing levels from baseline (mean level 1 year before digestive enzyme cartridge use) with levels while using the digestive enzyme cartridge at the 12‐ and 24‐month visits. At 12 months, there was a trend for an increase from baseline of 7.14 ng/ml (SD, 14.166; *P* = 0.086; *n* = 14).

## DISCUSSION

This retrospective case review examined the impact of long‐term use of a digestive enzyme cartridge on weight gain and GI symptoms in patients with CF receiving EN. In general, the digestive enzyme cartridge was well tolerated and resulted in a decrease in GI complaints among most participants. Interestingly, three patients were able to decrease their feeding volumes while using the digestive enzyme cartridge with overnight EN feeds, which may reflect the improvements in GI tolerance and absorption of nutrients. Previous reports of use of the digestive enzyme cartridge had demonstrated improved weight gain, but these were limited to only 3 months of treatment.^17^, ^18^ In our much longer observation, there was a positive effect on weight gain and other anthropometric measures throughout the period of analysis.

This study was limited by its very small sample size, which likely contributed to the decrease in mean WFL and BMI *z*‐scores after month 3. Another reason for the decrease can be attributed to inconsistent use of EN and the enzyme cartridge during the time of data collection. One patient was participating in feeding therapy and had their feeding volume decreased intermittently, one patient decreased their EN volume because of improved oral intake and lost weight as a result, and refill history on five patients showed interruptions in shipment of the cartridge to the patients because of an inability to confirm shipment with patients. Despite the small sample size in this study, we saw improvements in BMI, WFL, and BMI *z*‐scores and LFA and SFA *z*‐scores at some time points, as well as a decrease in GI complaints. This practical method of providing continuous fat hydrolysis during EN for patients with an already high treatment burden and multiple barriers to achieving adequate nutrition status is promising.

Previously, the only option for supplemental digestive enzymes with EN was capsules, which are better suited to bolus feeds in which the enzymes can mix with the food. Most PERT products are not designed or approved to be administered through a feeding tube, with few exceptions.^19^, ^20^ There are no prospectively published studies that evaluate PERT during enteral tube feeding, and this lack of evidence is noted in published guidelines for EN in patients with CF.^21^ The ability to provide continuous fat hydrolysis during enteral tube feeding offers a more practical method to maximize nutrition support in patients with CF. This case series provides supportive evidence that the digestive enzyme cartridge provides clinical benefit to undernourished patients receiving supplemental EN feeding.

## CONFLICT OF INTEREST

Patrick A. Flume has no financial relationship with Alcresta Therapeutics. Sara J. Hendrix is a member of the Alcresta Speakers Bureau and did not receive funding for her work on the manuscript. A. Archie Stone was an employee of Alcresta Therapeutics until December 2019, his work on this manuscript was initiated before his departure, and he no longer has any financial interests in Alcresta Therapeutics or its products. A. Archie Stone has two patents pending from his employment at Alcresta Therapeutics; neither are related to Relizorb or the data in this manuscript. Eric R. First reports personal fees from Alcresta Therapeutics, outside the submitted work. Mark Van Buskirk has no financial relationship with Alcresta Therapeutics.

## AUTHOR CONTRIBUTIONS

Sara. J. Hendrix contributed to the conception and design of the research and to the acquisition of the data; Mark Van Buskirk contributed to analysis of the data; Patrick A. Flume, A. Archie Stone, and Eric R. First contributed to the interpretation of the data and drafted the manuscript. All authors critically revised the manuscript, agree to be fully accountable for ensuring the integrity and accuracy of the work, and read and approved the final manuscript.
